# Topical Tranexamic Acid for Hemostasis of an Oral Bleed in a Patient on a Direct Oral Anticoagulant

**DOI:** 10.5811/cpcem.2020.1.45326

**Published:** 2020-03-27

**Authors:** Eric Boccio, Kyle Hultz, Ambrose H. Wong

**Affiliations:** * Yale School of Medicine, Department of Emergency Medicine, New Haven, Connecticut; † Yale-New Haven Hospital, Department of Emergency Medicine, New Haven, Connecticut

**Keywords:** Tranexamic acid, direct oral anticoagulant, TXA, DOAC, hemostasis

## Abstract

**Introduction:**

Tranexamic acid (TXA) is an antifibrinolytic agent currently approved and utilized in the treatment of dysfunctional uterine bleeding, traumatic extracranial hemorrhage, anterior epistaxis, and dental procedures on patients with hemophilia. There is a paucity of literature evaluating the use of TXA for hemostasis in patients on direct oral anticoagulants (DOACs).

**Case Report:**

Our patient, a 72 year-old male on rivaroxaban, presented with persistent bleeding following a punch biopsy of the buccal mucosa. Given the site of bleeding, inability to effectively tamponade, patient’s anticoagulated state, and risk of impending airway compromise, a dressing was soaked with 500 milligram (mg) of TXA and was held in place with pressure using a makeshift clamp until a thrombus formed. Hemostasis was achieved preventing the need for acute ENTotolaryngologic intervention and/or intubation. The patient was observed in the medical setting overnight and discharged home without any recurrence of bleeding or adverse events.

**Discussion:**

This case report describes our experience achieving hemostasis for an otherwise uncontrollable oral bleed in an anticoagulated patient on a DOAC who could not be reversed. Intervention is simple to perform, cost-effective, and requires few resources which are readily available in most emergency departments.

**Conclusion:**

We report a novel application of TXA to control an oral mucosal bleed in an anticoagulated patient which was on a DOAC refractory to traditional measures.

## INTRODUCTION

Tranexamic acid (TXA) is an antifibrinolytic agent approved by the Food and Drug Administration for the treatment of cyclic, heavy menstrual bleeding and prevention of hemorrhage during tooth extractions in patients with hemophilia.^1^ Recently, TXA use has expanded rapidly for a variety of off-label indications and is now considered the antifibrinolytic of choice in patients who present to the emergency department (ED) with extracranial hemorrhage following a traumatic injury.^2^ Further analysis of the CRASH-2 (Corticosteroid Randomization after Significant Head Injury) trial demonstrated a significant effect of TXA on favorable functional outcomes for the lowest-risk (<6% mortality) group. Due to its cost-effectiveness and exceptional safety profile TXA is recommended for patients presenting within three hours of sustaining a traumatic injury.^3^

Atomized nasal TXA with external compression was proven to be as effective as standard nasal packing at hemostasis, superior at preventing rebleeding events within 24 hours, and more comfortable than anterior nasal packing and traditional external compression in managing anterior epistaxis.^4^ The use of TXA, both systemically and topically, in the setting of dental extraction and orthognathic surgery has been documented in the literature.^5^ The use of TXA suspension-soaked gauze, made from crushed 500 milligram (mg) TXA tablets and water, applied directly to the extracted cavity to prevent bleeding has been successful in hemophilia patients undergoing dental procedures.^6^ The use of a 0.05% TXA irrigation solution following orthognathic surgery and 5% TXA mouthwash administered after gingival manipulation and scaling in patients with hemophilia has also been shown to be effective in prevention and/or treatment of clinically significant oropharyngeal bleeding.^7^–^8^

It is important to note, however, that each of these dosage forms requires manipulation of commercially available TXA products, and may not be ideal for urgent use in the ED setting. A recent Cochrane Review examined randomized controlled trials of people on continuous treatment with vitamin K antagonists (VKA) or direct oral anticoagulants (DOAC) undergoing oral or dental procedures using antifibrinolytic agents to prevent perioperative bleeding.^9^ Although the studies demonstrated a beneficial effect of locally applied TXA for patients on continuous VKAs, no eligible trials in people on continuous treatment with DOACs were identified.

## CASE REPORT

A 72 year-old-male with known past medical history inclusive of right lower extremity deep vein thrombosis currently on the DOAC rivaroxaban, myelodysplastic syndrome status post stem cell transplant complicated by graft versus host disease (GVHD), hyperlipidemia, stage three chronic kidney disease, and type II non-insulin dependent diabetes mellitus presented to the ED with right buccal mucosa bleed after undergoing punch biopsy by dermatology six hours prior. The last administered dose of rivaroxaban was earlier the same morning, as he was not instructed to hold the dose because the diagnostic punch biopsy for GVHD workup was scheduled ad hoc while he was in the hospital for routine follow-up. Despite manually holding direct pressure with a paper towel over the bleeding site, the patient stated that he had been unable to achieve hemostasis, which was affecting his ability to speak and sleep due to the continuous need to spit out blood, prompting his visit to the ED. A single, folded, paper towel sheet became saturated and required exchange every 15 minutes.

**CPC-EM Capsule**

What do we already know about this clinical entity?

Tranexamic acid (TXA) clinical applications include menstrual bleeding, dental extractions, anterior epistaxis, and traumatic extracranial hemorrhage.

What makes this presentation of disease reportable?

Emergency department patients on direct oral anticoagulants (DOAC) may present with bleeding, which is refractory to traditional measures.

What is the major learning point?

TXA was applied topically with no major adverse effects to achieve hemostasis of an oral bleed in a patient on DOAC who could not be reversed.

How might this improve emergency medicine practice?

Further investigation into the use of topical TXA as a treatment option for refractory hemorrhage in patients on DOACs is warranted.

Review of systems was notable for oral bleeding, minor post-procedural pain (2/10 in severity) at the biopsy site, and three days of acute on chronic constipation, believed to be unrelated to his chief complaint. The patient denied shortness of breath, cough, hemoptysis, and dysphagia. On physical examination, the airway was intact, and there was an active, continual oozing of dark blood from a five-millimeter biopsy site along the right buccal mucosa. Due to the duration of bleeding and reported amount of saturated dressings, a complete blood count, general chemistry panel, and coagulation panel were drawn, which was notable for a hemoglobin and hematocrit of 12.9 grams per deciliter (g/dL) [14–17.4g/dL] and 37.6% [42–54%], respectively, with prothrombin time and international normalized ratio of 10.1 seconds [9.2–11.9 seconds] and 0.91 [0.8–1.2], respectively. The patient was placed in an examination room with continuous wall suctioning and given a Yankauer suction tip to clear his oropharynx.

**Image 1:**
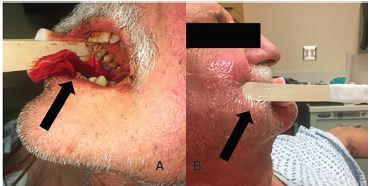
(a)Tranexamic acid-soaked dressing held in placed with tongue depressors and (b) adhesive dressing over patient’s cheek.

**Image 2:**
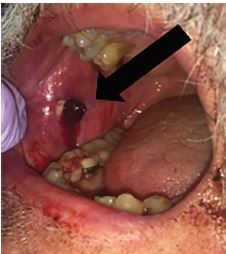
Visualized thrombus over punch biopsy site after being treated with pressure dressing soaked in 500 milligrams of tranexamic acid and held in place for 30 minutes.

Given the wide base of the biopsy site and lack of overlapping tissue, the wound was not amenable to hemostasis through placement of sutures. Thus, along with emergency pharmacy guidance and approval, a sterile 4-inch × 4-inch gauze dressing was soaked with 500 mg [5 milliliter (mL)] of 100 mg/mL TXA and placed over the bleeding site. The soaked gauze was held in place with pressure applied by two tongue depressors taped on one end with medical tape and held to the patient’s cheek with an adhesive dressing (Images 1A and 1B). After 30 minutes, the device and gauze were removed, and a clot was visualized (Image 2). Due to the possibility of recurrent bleeding and impending airway compromise, the patient was admitted under the otolaryngology service for observation and was discharged from the hospital eight hours later without any significant rebleeding events. There was no 72-hour ED return visit noted within the health system’s electronic health records.

## DISCUSSION

TXA is a lysine analog similar in chemical structure to aminocaproic acid, but with 10 times more potency in its affinity to both the strong and weak receptor sites of the plasminogen molecule. By competitively binding to plasminogen, TXA effectively blocks the conversion of plasminogen to plasmin, thereby preventing fibrinolysis and, in turn, promotes stabilization of the fibrin clot.^10^ The excellent safety profile of TXA is likely twofold: therapeutic doses do not cause platelet aggregation in vitro, and when applied topically there is little to no systemic absorption. In light of the proposed mechanism and known safety profile, we suggest further investigation into the use of topical TXA as a potential first-line or adjunctive treatment option for difficult to control or refractory hemorrhage in particular instances.

## CONCLUSION

This case report addresses a novel application of TXA, a medication that has become increasingly popular in the ED setting to address a very prevalent issue among our anticoagulated patient population on DOACs, specifically achieving hemostasis that is refractory to more traditional measures. We describe our experience achieving hemostasis for an otherwise-uncontrollable oral bleeding event in a patient whose anticoagulated state could not be reversed. Our intervention is simple to perform and cost-effective (approximately $21.10 US dollars per 100mg/mL 10mL vial). It requires very few resources, which are readily available in most EDs and, most importantly, appears effective. We believe our methods are widely generalizable and can be easily assimilated into the armamentarium of emergency medicine providers in a wide range of clinical environments.
